# Retraction: Photo-responsive hole formation in the monolayer membrane wall of a supramolecular nanotube for quick recovery of encapsulated protein

**DOI:** 10.1039/d4na90110j

**Published:** 2024-10-17

**Authors:** N. Kameta, Y. Kikkawa, Y. Norikane

**Affiliations:** a Nanomaterials Research Institute, Department of Materials and Chemistry, National Institute of Advanced Industrial Science and Technology (AIST) Tsukuba Central 5, 1-1-1 Higashi Tsukuba Ibaraki 305-8565 Japan n-kameta@aist.go.jp +81-29-861-4545 +81-29-861-4478; b Research Institute for Advanced Electronics and Photonics, Department of Electronics and Manufacturing AIST, Tsukuba Central 5, 1-1-1 Higashi Tsukuba Ibaraki 305-8565 Japan

## Abstract

Retraction of ‘Photo-responsive hole formation in the monolayer membrane wall of a supramolecular nanotube for quick recovery of encapsulated protein’ by N. Kameta *et al.*, *Nanoscale Adv.*, 2022, **4**, 1979–1987, https://doi.org/10.1039/D2NA00035K.

We the named authors hereby wholly retract this *Nanoscale Advances* article due to the fact that the paper has wrong electron microscopy images in Fig. 2d and e on the part of the first author, who is affiliated with the National Institute of Advanced Industrial Science and Technology (AIST).

Fig. 2d and e had incorrect scale bars, which were approximately 2 times longer than the actual ones. Such errors in the scale bars would be inconsistent with the description of the sizes of the diameters and the wall thicknesses of the nanotubes.

The authors respectfully retract this paper, because these events were determined to amount to scientific misconduct and the retraction of this paper was recommended by AIST. AIST verified that the first author was responsible for the misconduct and no other co-authors were engaged in them.

Signed: Yoshihiro Kikkawa, Yasuo Norikane, Naohiro Kameta

Date: 3rd October 2024

Retraction endorsed by Jeremy Allen, Executive Editor, *Nanoscale Advances*.

